# Massively parallelized brain tractography using compute clusters, supercomputers, and graphics processing units

**DOI:** 10.1162/IMAG.a.1341

**Published:** 2026-08-19

**Authors:** John Kruper, Ariel Rokem

**Affiliations:** Department of Psychology, University of Washington, Seattle, WA, United States; Krembil Centre for Neuroinformatics, Campbell Family Mental Health Research Institute, Centre for Addiction and Mental Health, Toronto, ON, Canada; Department of Psychiatry, University of Toronto, Toronto, ON, Canada

**Keywords:** tractography, graphics processing units, high performance computing, diffusion MRI

## Abstract

Tractography based on diffusion-weighted MRI (dMRI) is the predominant *in vivo* method for mapping the brain’s white matter. However, it is also one of the most computationally demanding steps in neuroimaging data analysis—requiring the generation and filtering of millions of streamlines per subject. Over the past decade, high-performance computing (HPC) and graphics processing units (GPUs) have reshaped the landscape of tractography. What once required hours or days per subject can now be performed in minutes or even seconds. This facilitates faster processing of large-scale studies and high-resolution datasets, supports real-time clinical applications, and enables more computationally demanding algorithms. This review surveys the recent wave of applications of HPC and GPUs to massively parallelize tractography. We discuss how these advances have accelerated tractography pipelines, identify common strategies for parallelization, and highlight opportunities where further parallelization could improve efficiency and accuracy.

## 1 Introduction

The brain is a highly interconnected system, and its function depends on the coordinated activity of spatially distributed neural populations. Communication between different brain regions depends on the integrity of the white matter, the component of the brain composed primarily of bundles of myelinated axons that form the long-range connections of the nervous system. These myelinated fibers act as biological wiring, enabling rapid and efficient transmission of electrical signals between brain areas. Alterations in white matter microstructure have been linked to a variety of disorders, and white matter plasticity has been associated with learning (Fields, 2008; McKenzie et al., 2014; Sampaio-Baptista & Johansen-Berg, 2017; Xin & Chan, 2020). Therefore, understanding the structural organization and physical properties of the white matter is crucial for understanding the brain as a whole.

Tractography reconstructs estimates of the 3D trajectories of white matter tracts. It is the primary *in vivo* method for mapping the brain’s white matter and quantifying the microstructural properties of major white matter pathways. Tractography is important because it enables tract reconstruction in subject space, reducing the reliance on atlas-based priors. Second, it permits finer-grained investigation than whole-bundle atlas analyses. Third, it supports connectivity-based analyses that are not accessible through voxel-based metrics alone. However, tractography can be computationally intensive.

Each tractography reconstruction may require generating millions of candidate pathways, fitting complex models to tens of thousands of voxels, or optimizing parameters across the entire white matter. Human *in vivo* tractography uses diffusion-weighted Magnetic Resonance Imaging (dMRI). Datasets that include dMRI measurements have grown from tens to thousands of subjects and from millimeter to submillimeter spatial resolutions, and at the same time, dMRI pipelines have become increasingly computationally demanding (Alfaro-Almagro et al., 2018; Casey et al., 2018; Setsompop et al., 2018; Van Essen et al., 2012; Wang et al., 2021). Without massive parallelization, these steps can take hours to days per subject, making large-scale studies difficult and interactive studies infeasible.

High-performance computing (HPC) and graphical processing units (GPUs) provide practical means to address these computational demands. By massively parallelizing computation across many central processing units (CPUs) or thousands of GPU cores, tractography workflows that once required hours or days can now run in minutes. These systems have been increasing in availability (Abid, 2016; Madhyastha et al., 2017; Parnell et al., 2019; Shu et al., 2025; Yogatama et al., 2025), presenting opportunities for new applications of tractography. The present review takes stock of the state of massively parallelized tractography. We survey the software ecosystems and computational approaches used for massive parallelization. Finally, we identify open challenges and directions for future research.

Throughout this review, we report performance using raw execution times rather than relative speedups to discourage direct comparisons. While these timings offer a useful approximation of performance, they often represent idealized scenarios that may omit scheduling overhead or I/O bottlenecks. Different labs will have access to different hardware and have different resource constraints. Furthermore, comparing timings directly is challenging given the diversity of hardware and infrastructure used throughout this review, as well as heterogeneous reporting standards. We also provide guidance on best practices for benchmarking approaches that will facilitate future comparisons across methods.

## 2 The Case for Massively Parallel Tractography

Tractography is computationally demanding. To understand why, it is useful to distinguish between the two major classes of tractography algorithms. These methods can be broadly categorized into local and global frameworks, with local approaches constituting the prevailing paradigm in current practice.

Local algorithms depend on generating a large number of candidate paths through the white matter, called streamlines. Each streamline is meant to estimate a plausible trajectory of a group of axons. These algorithms are typically serial and greedy, where each step is computed independently. Hence, compute time scales with the number of streamlines and inversely with the size of each step. However, the ideal number of streamlines depends on the use case. In connectomics—the construction of brain networks from tractography—increasing the number of streamlines creates a more dense tractography, changing the resulting network properties in ways that are not necessarily more accurate (Jung et al., 2021). Nevertheless, in Human Connectome Project (HCP) data (Van Essen et al., 2012), increasing streamlines increased reproducibility, with a minimum of one million streamlines required for excellent reproducibility (intraclass correlation coefficient higher than 0.75) (Roine et al., 2019). Ultimately, given the inherent uncertainty in measuring connectivity between regions with low connection probabilities, in some cases, even 20 million streamlines may prove insufficient (Aeschlimann et al., 2025). In white matter tract recognition, increasing the number of streamlines increased coverage of white matter pathways. Furthermore, a previous study found that the minimum number of streamlines required to achieve reliable microstructural measurements depends on the tract and measurement (Reid et al., 2020). Thus, the minimum number of streamlines depends on the dataset, use case, and tract of interest. Generally, millions of streamlines are necessary to reconstruct deep white matter tracts (Roman et al., 2019), with common defaults ranging from 1 to 10 million (Kruper, Richie-Halford, et al., 2025;  Tournier et al., 2019; Wasserthal et al., 2018). Counterintuitively, smaller tracts may require even more streamlines, because they are difficult to delineate (Kruper & Rokem, 2023).

Global methods can be even more computationally intensive. Unlike local approaches, which generate streamlines independently, global tractography treats tractography as a single optimization problem. These methods define a global objective function over all voxels and all candidate pathways, and then perform iterative fitting to jointly estimate a configuration of streamlines that best explains the measured diffusion signal. A large number of iterations are required, with one popular algorithm defaulting to 1 billion iterations (Christiaens et al., 2015).

Regardless of the approach, generating a tractography that is robust to noise and covers complex fiber configurations requires time-consuming computations. Currently, tractography is performed most frequently on single CPUs. HPC and GPUs represent massively parallel alternatives. There is an increasing number of applications of tractography where massive parallelization is necessary. These broadly break down into four categories:

1. **Datasets with a large number of subjects**. At least four studies are in the process of collecting more than 10,000 scans, including: Adolescent Brain Cognitive Development (ABCD) (Karcher & Barch, 2021); UK Biobank (Alfaro-Almagro et al., 2018); HEALthy Brain and Child Development (HBCD) Study (Volkow et al., 2024); and Rhineland study (Breteler et al., 2014). In these cases, software written specifically for HPC is typically not necessary, as subjects can be processed independently on separate nodes. However, depending on availability and cost, GPU acceleration can reduce processing time significantly. As an example, Hernandez-Fernandez et al. (2019) showed that to fit NODDI (neurite orientation dispersion and density imaging (H. Zhang et al., 2012); a popular biophysical model) on a UK Biobank subject, the fastest CPU fit takes 6 hours and 45 minutes with 72 cores, while a single GPU can fit it in less than 7 minutes (Hernandez-Fernandez et al., 2019). Similarly, in Kruper et al. (2023), GPU-accelerated tractography was used to recognize the optic radiations, a visual white matter tract, in 7,438 subjects from the UK Biobank (Rokem et al., 2021), reducing average tractography time per subject from 1.5 hours to just under 30 seconds. Scaling these speedups to 100,000 subjects reduces the compute time to fit NODDI on all UK Biobank subjects by 75.9 years to 1.3 years, and the time to recognize the optic radiations by 17.1 years to 1.14 months. These correspond to reductions of approximately 98.3% and 99.4%, respectively.

2. **High resolution measurements**. Submillimeter-resolution *in vivo* dMRI datasets that have been published in recent years (H.-C. Chang et al., 2015; Dong et al., 2025; Haldar et al., 2020; Movahedian Attar et al., 2020; Ramos-Llordén et al., 2020; Setsompop et al., 2018; Wang et al., 2018) enable the identification of smaller but clinically important white matter tracts (Rıós et al., 2022). Among these, the dataset from Wang et al. (2021), at 760 *μ*m isotropic resolution, is notable for being publicly available, providing a benchmark for further algorithmic development. The high spatial resolution of these data greatly increases computational demands; for example, the raw k-space data acquired by Wang et al. is approximately 7 terabytes in size. These datasets are useful for corresponding dMRI to higher-resolution modalities like microscopy, and for moving towards building a mesoscale connectome (Heilbronner et al., 2025). Global tractography has already been applied to submillimeter dMRI data using a supercomputer (Legeay, 2025). At these resolutions, conventional approaches can be computationally prohibitive.

3. **Increased algorithmic complexity**. Massively parallelized tractography opens the door to previously computationally infeasible approaches. Global tractography algorithms have long been constrained by computational limits, forcing trade-offs between feasibility and model complexity (Christiaens et al., 2015; Mangin et al., 2013). Additionally, local tractography algorithms that attempt to integrate information across nearby voxels to determine step direction could also benefit from acceleration (Aydogan & Shi, 2021; Girard et al., 2014).

4. **Interactive tractography**. Finally, GPU acceleration enables real-time interactive tractography, which is particularly valuable in clinical contexts. Tractography supports preoperative assessment in brain tumor patients (Berman et al., 2004) and helps localize targets for deep brain stimulation (Calabrese, 2016). In both cases, user-defined regions of interest (ROIs) guide which streamlines are generated or retained. Because of individual variability, tractography must often be regenerated in real-time as ROIs are adjusted by the clinician, which requires efficient GPU-based methods (Mittmann et al., 2008; Petrovic et al., 2007; Wu et al., 2025).

### 2.1 The scope of the present review

**Fig. 1.**
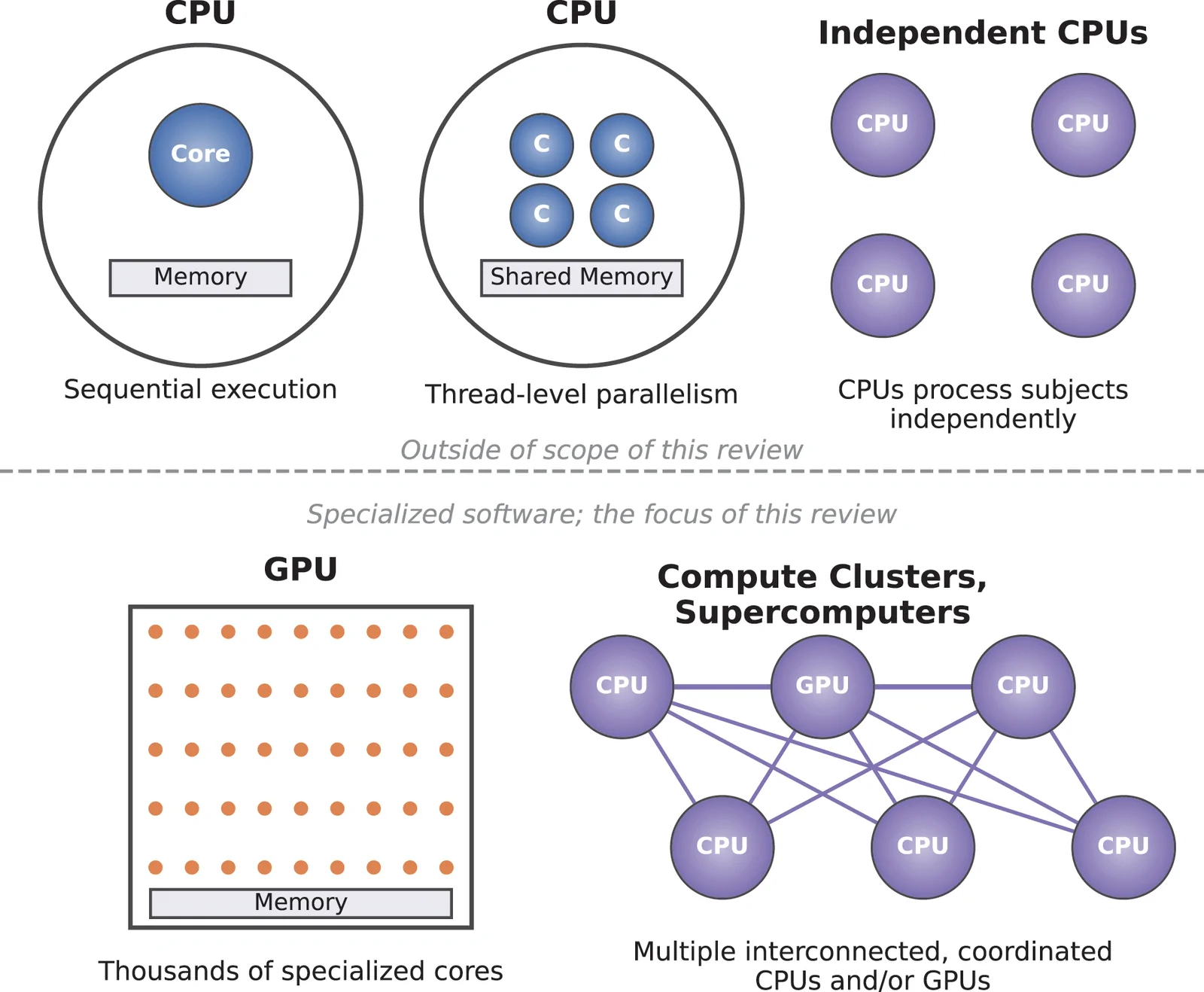
Illustration of different hardware platforms. The upper panel depicts single-core and multicore CPU architectures, with either one core or multiple cores working on a single task. These configurations characterize most desktop computing environments. Also shown are CPUs in a cluster working on tasks independently. The configurations shown above the dotted horizontal line do not require HPC-specific or GPU-specific software and are not the primary focus of this review. The lower panel shows the transition to GPUs and distributed compute clusters, which represent massively parallel architectures. These high-performance computing environments enable large-scale data processing and motivate the development of specialized HPC and GPU software that differs fundamentally from traditional single-CPU implementations. See Section 3 for more details on different hardware platforms.

This review focuses on software developed for GPUs and HPC environments (see the lower panel of Fig. 1). We refer to this software as massively parallel generally, or GPU-accelerated when referring specifically to GPU implementations. Many tractography software libraries focus on typical desktop computers with one CPU as the target hardware (González Rodrıǵuez et al., 2024;  Tournier et al., 2019; Yeh, 2025). With these, researchers can still take advantage of multiple CPUs on compute clusters (or even on supercomputers; Jimbo et al., 2023) by parallelizing across study participants. In addition, some software is written to more explicitly take advantage of multiple cores via multithreading. There are also purely algorithmic advances which can yield similar speedups (Caiafa & Pestilli, 2017; Daducci, Canales-Rodrıǵuez, et al., 2015). Taken together, single-CPU computing can be efficient enough for many common tasks and datasets. Nevertheless, to maintain focus, this review does not include software with only single-CPU implementations.

Furthermore, this review focuses on tractography-related computations. Efforts to massively parallelize other dMRI computations unrelated to tractography, such as eddy current correction, are outside of the scope of this review (Maller et al., 2020). There are also efforts to use neural networks or deep learning to enhance or replace elements of the tractography pipeline (Legarreta et al., 2021; Théberge et al., 2021). These neural networks are often trained and evaluated using GPUs. However, these advances belong to a distinct category and have already been covered by recent reviews (Barati Shoorche et al., 2025; Faiyaz et al., 2023; Karimi & Warfield, 2024).

For relevance, the remainder of this review focuses only on work from 2015 onward. The first GPU implementation of both orientation distribution function (ODF) estimation and tractography was published in 2007 (McGraw & Nadar, 2007). Although it was recognized that fiber tracking is naturally parallelizable across streamlines, using GPUs for tract generation did not become commonplace (Eklund et al., 2013). Instead, the focus was on using GPU acceleration to enable interactivity and rapid visualization (Mittmann et al., 2008; Petrovic et al., 2007). Users would interactively select regions of interest, and tractography could be generated and visualized in seconds. This interactive use case persists today (Wu et al., 2025), but the present review focuses on tractography generation and filtering and does not cover software that only performs visualization.

## 3 Hardware

Scaling tractography requires exploiting the modern computing ecosystem. This ecosystem can be thought of as a hierarchy. At the base, tier 0, are single CPU systems running programs in serial or using multi-threading. Parallelized algorithms on this tier are outside the scope of this review. Next is tier 1, where additional hardware, mainly GPUs, is used to complement the CPUs. In tier 2 are mid-scale compute clusters. These consist of multiple interconnected computers, each with its own CPUs and potentially GPUs, working in tandem. Finally, in tier 3 are supercomputers and large-scale compute clusters. Understanding the strengths and limitations of each tier is important for effective application to tractography pipelines.

### 3.1 Tier 1: GPUs

Computational architectures can be categorized by the number of instruction streams and data streams they support (Flynn, 1972). Modern CPUs are multiple instruction, multiple data (MIMD): there are several cores, and each core can follow its own path through a program and handle a wide variety of tasks at once. This flexibility makes CPUs good at “branch-heavy” workloads—situations where the program takes one of many possible paths through the code, like following an if/else branch.

GPUs, by contrast, are designed for single instruction, multiple data (SIMD) execution. They schedule execution in thread-groups (often 32 threads). Within a thread-group, threads must execute the same instruction at the same time to be parallel. If threads diverge (as in an if statement), execution serializes, reducing efficiency. GPU memory access is characterized by very high bandwidth but also high latency. Performance depends heavily on coalesced memory accesses, where threads in a thread-group access consecutive locations in memory. Thus, GPUs are most efficient when performing uniform operations on adjacent memory, evident by their advantages in linear algebra.

Programming GPUs directly is most often done through CUDA, NVIDIA’s proprietary parallel computing platform. CUDA provides extensions to C/C++ and Python that expose GPU hardware primitives. Beyond CUDA, many languages support multiple hardware backends with a single codebase. Of these, the most popular in tractography is OpenCL (Stone et al., 2010). It is a widely-used open standard designed to run across GPUs, CPUs, and other accelerators, and it is accessible from Python via PyOpenCL (Klöckner et al., 2012). Other notable interfaces include HIP (commonly used for AMD GPUs) and ArrayFire (Malcolm et al., 2012). These ecosystems illustrate the balance between portability and performance: CUDA is efficient and mature, with widespread use, but requires NVIDIA hardware, while frameworks like OpenCL aim to support multiple vendors at the cost of some abstraction overhead.

### 3.2 Tier 2: Mid-scale compute clusters

Compute clusters consist of interconnected computers, called nodes. Each node contains its own processors (CPUs and sometimes GPUs), memory, storage, network interface, and operating system. Nodes do not share memory directly: they communicate with other nodes over the cluster’s high-speed interconnect. Multiprocessing is when multiple nodes are used to complete tasks in parallel. For tractography, subjects are often processed independently across nodes, which is straightforward and does not require HPC-specific software. However, one can also use multiprocessing to perform computationally expensive algorithms on a single subject by parallelizing across nodes. The Message Passing Interface (MPI) (Walker & Dongarra, 1996) is a standard API for sending and receiving messages between processes on different nodes.

In contrast, multithreading is a parallel programming model in which a single process creates multiple threads (sequences of instructions) that run concurrently on different cores of the same node (typically within the same CPU) and operate on a shared memory space. Multithreading can be programmed with OpenMP, an API that provides compiler directives to parallelize loops and other constructs across multiple cores (Dagum & Menon, 1998). OpenMP is most often used in C, C++, and Fortran—the core languages of high-performance computing. For many researchers, however, Python is a more comfortable language for prototyping. Fortunately, there are ways to bridge Python with OpenMP. For example, Cython—a superset of Python with static typing—allows direct insertion of OpenMP pragmas (Behnel et al., 2010). Another option (still in its early stages) is pyOMP (Mattson et al., 2021), a Python interface to OpenMP that provides decorators for specifying parallel regions.

Because OpenMP cannot directly span across nodes, it is complementary to MPI, which handles inter-node communication. More recently, higher-level workflow and programming frameworks such as Parsl and Kokkos have emerged to help researchers use multithreading and multiprocessing in tandem (Babuji et al., 2019; Cai et al., 2024; Edwards et al., 2014). Parsl is a Python parallel scripting library that lets users annotate Python functions (or external commands) as apps and then automatically orchestrates their execution as a dataflow graph through the compute cluster or supercomputer. Kokkos is a C++ performance-portability library that provides abstractions for both parallel execution and memory management, enabling developers to write a single codebase that can target backends like the aforementioned CUDA, HIP, or OpenMP.

### 3.3 Tier 3: Supercomputers and large-scale compute clusters

Supercomputers represent the largest scale of high-performance computing, extending the principles of compute clusters to tens of thousands of nodes connected by specialized high-bandwidth, low-latency interconnects. Like clusters, they consist of many individual CPUs and GPUs, but their defining characteristic is scale. Modern systems achieve performance measured in exaflops (one quintillion floating-point operations per second) (Schneider, 2022), with enormous memory bandwidth and I/O systems engineered to keep up with the demands of extreme parallelism. Supercomputers are designed to solve problems so large that neither a single GPU nor a conventional cluster can complete them in a reasonable time.

## 4 Recent Applications

Tractography is a multi-step process, described in detail in previous reviews (e.g., Jeurissen et al., 2019). This section is organized according to these stages: First, we discuss reconstructing ODFs within each voxel. Next, tractography generation is divided into local and global or geodesic approaches. Local methods trace streamlines step-by-step from seed points using local orientation estimates, whereas global and geodesic methods infer the configurations of multiple fibers simultaneously as optimization problems. Finally, we consider tract filtering, which refines or weights the resulting tractography to improve its validity and interpretability.

### 4.1 ODF reconstruction

Within each voxel of diffusion MRI, the diffusion signal is thought to reflect the distribution of water displacements along different orientations. To model this, researchers estimate an ODF that describes how strong diffusion is in different orientations.

Within ODF models, three broad families exist. Biophysical models explain the diffusion using multiple signal components informed by the known structure of the white matter (e.g., sticks to represent fiber populations, balls to represent extracellular space). These methods provide interpretable biophysical quantities, such as axonal volume fractions or orientation dispersions. However, they often require computationally demanding non-linear optimization to fit to the data (H. Zhang et al., 2012).

Signal-representation models, in contrast, fit the diffusion signal directly using mathematical bases such as spherical harmonics or tensors. The most prominent example is diffusion tensor imaging (DTI), which models diffusion as a single 3D Gaussian distribution. The tensor representation is computationally efficient and widely adopted, but it cannot resolve multiple fiber orientations within the same voxel. Higher-order tensor models and spherical harmonic representations extend this idea by allowing more complex angular structure. Constrained spherical deconvolution (CSD), in particular, estimates sharper ODFs by deconvolving the signal with a response function that represents a single coherent fiber population. Extensions such as multi-shell, multi-tissue CSD (MSMT-CSD) fit multiple response functions simultaneously (Jeurissen et al., 2014), enabling improved modeling of voxels containing mixtures of white matter, gray matter, and cerebrospinal fluid.

Finally, the third family consists of Bayesian models, which quantify the posterior orientation uncertainty of cross-fiber populations. Unlike other approaches, these models provide uncertainty estimations for model parameters. There are experimental conditions where Bayesian models outperform other approaches, and vice versa (Daducci et al., 2014). Bayesian models can be computationally expensive to fit, making them suitable targets for massive parallelization (Hernández et al., 2013).

Most ODF reconstruction methods operate at the voxel level: each voxel’s diffusion signal is modeled independently of its neighbors. This makes the problem embarrassingly parallel–the same reconstruction procedure can be applied to many voxels in parallel without communication between them. However, many ODF models are also rapidly fit. DTI is typically linearized for technical simplicity and then solved with weighted least squares, making it tractable on standard CPUs without parallelization, even on large datasets (Salvador et al., 2005; Veraart et al., 2013). Thus, the role of GPUs and HPCs is confined to more complicated models and solvers (L.-C. Chang et al., 2014).

Once a model has been fit, it can be used in two primary ways. First, it can calculate fiber ODFs (fODFs) to guide tractography. Signal-representation and Bayesian models are the most popular choices for this role. Second, models can estimate tissue properties for downstream analysis. Biophysical models provide interpretable values, though signal-representation and Bayesian models are also frequently used. In this section, we will cover models from both use cases.

As shown in Table 1, a series of recent works have applied HPC and GPU frameworks to massively parallelize ODF reconstruction. The first implements RUMBA-SD (Robust and Unbiased Model-Based Spherical Deconvolution), a signal-representation approach that explicitly models the non-Gaussian noise present in multichannel MRI (Rician and noncentral chi-squared) (Canales-Rodrıǵuez et al., 2015). The estimation is posed as a maximum-likelihood problem with optional spatial regularization, and solved using an iterative Richardson–Lucy–style expectation–maximization scheme. Garcıá Blas et al. (2016) used hardware agnostic C++ libraries to remain portable and flexible across heterogeneous systems. They fit RUMBA-SD on 983,040 voxels with 100 directions each in under 50 seconds using the NVIDIA GTX 680.

**Table 1.** ODF model reconstruction, parallelized on GPUs and HPC systems, sorted by year, since 2015.

Authors	Year	Model	Tier	Tool(s)
Garcıá Blas et al.	2016	RUMBA-SD	1, 2	ArrayFire, OpenMP
Harms et al.	2017	Multiple	1, 2	OpenCL
Hernandez-Fernandez et al.	2019	Multiple	1	CUDA
Poirier & Descoteaux	2024	a-ODF	1	OpenCL
Xu et al.	2025	Multiple	1	MATLAB

Harms et al.’s (2017) work followed this by introducing a framework for robust, fast, general nonlinear optimization implemented in OpenCL. The software supports arbitrary combinations of compartment models, likelihood functions, and optimizers, which are automatically compiled at runtime. These multiple compartments are used to fit a variety of models (such as NODDI), which require slower iterative solvers such as Levenberg–Marquardt or Nelder-Mead Simplex. When multiplied across millions of voxels, these costs dominate runtime. Previously, AMICO was proposed to re-formulate biophysical model fitting as a linear problem. However, AMICO assumes a zero-mean Gaussian distribution for the noise structure, which is likely to be incorrect (Daducci, Canales-Rodrıǵuez, et al., 2015). GPU acceleration is therefore especially impactful in this setting, where parallelism over voxels combines with highly arithmetic inner loops to deliver substantial speedups without reformulation. GPUs can process each voxel in a different subgroup, then use SIMD (see Section 3.1) within each subgroup to parallelize model fitting. Harms et al. found that the gradient-free Powell conjugate-direction algorithm outperformed the other algorithms in run time, accuracy, and precision. They fit NODDI in 5 minutes and 32 seconds on their example subject (1.5 mm isotropic; 552 volumes).

Next, Hernandez-Fernandez et al. (2019) introduced a pair of CUDA-based frameworks that extended GPU acceleration to both voxel-wise Bayesian modeling and whole-brain tractography. The tractography component is discussed further in Section 4.2. The modeling component is named the CUDA Diffusion Modelling Toolbox (cuDIMOT). cuDIMOT builds upon earlier work that focused exclusively on Bayesian inference for the ball-and-stick model (Bedpostx) (Hernández et al., 2013). The updated framework provides a flexible front end for user-defined diffusion models, which can be fit using either Levenberg–Marquardt optimization or Markov chain Monte Carlo (MCMC) sampling—similar in concept to the OpenCL-based framework of Harms et al. (2017). However, the implementation here is CUDA-specific, yielding high performance on NVIDIA GPUs at the expense of portability across hardware platforms (see Section 3.1). On average, a single K80 NVIDIA GPU fits NODDI on one UK Biobank subject in 6.8 minutes, and Ball and 2 sticks with a gamma-distribution for the diffusivity in 11.3 minutes. Subsequent validation confirmed that the algorithm yields results consistent with the CPU version (Kim et al., 2022).

Moving towards signal-representation models, the work by Poirier and Descoteaux introduced a unified filtering approach for estimating asymmetric orientation distribution functions (a-ODFs) (Poirier & Descoteaux, 2024). Typically, orientation distribution functions have antipodal symmetry. However, a-ODFs relax this constraint to enable the representation of bending, branching, and fanning fiber configurations within a single voxel. These can be reconstructed directly from the signal (similar to other ODFs), or through filtering methods, which derive a-ODFs by applying spatial–angular filters to previously reconstructed symmetric ODFs. Whereas previous methods specialized in distinct regularization schemes (e.g., Gaussian cones, bilateral filters, or cone-beam regularization), this study systematically reformulated them into a single generalized filtering equation that integrates spatial, angular, alignment, and intensity-based weighting terms. It has both an OpenCL for Python and Numba version (Klöckner et al., 2012; Lam et al., 2015), which each provide an effective balance between accessibility and performance. Filtering a full ODF volume from an HCP subject (1.25 mm isotropic) took about 65 seconds on an NVIDIA GTX1660 GPU. The resulting a-ODFs can be used for tracking or to quantify asymmetric fiber configurations.

Xu et al. used MATLAB’s gpuArray to create a general-purpose toolbox for ODF reconstruction (Xu et al., 2025), which includes a variety of reconstruction techniques, including non-linear least squares, Bayesian approaches, machine learning, and dictionary matching methods. The methods are all GPU-accelerated and accessible via a graphical user interface (GUI), to make customization accessible to users with limited programming experience. Using the NVIDIA GeForce RTX 4070 GPU and dictionary mapping (a reconstruction technique where signals are matched to a precomputed dictionary), they reconstructed the ODFs of 100,000 voxels in under 5 seconds.

### 4.2 Local tractography

After reconstructing the ODFs, streamlines can be generated by starting from a seed point and advancing step by step through the ODF field. At each step, the next direction is chosen based on a local ODF peak, and the process continues until a stopping criterion is met, such as entering gray matter. This process traces a single plausible path of a group of axons. Local tractography reconstructs white matter connectivity by generating many such streamlines.

Streamlines are first generated *deterministically* by always following the largest local direction, with limits on how sharply they can turn in one step (a maximum turning angle). While in deterministic tractography, the path is deterministic given the starting seed location, probabilistic algorithms do not follow the largest local peak to take a next step; instead, they choose the next step direction *probabilistically*, weighted by peak size. The process can be viewed as a Monte Carlo sampling problem: each streamline samples from the distribution of possible orientations rather than following a single deterministic maximum. This flexibility samples fiber crossings, branchings, and low–signal-to-noise regions more widely. The implausible streamlines are filtered away in subsequent tract filtering steps. However, the benefits of probabilistic tractography rely on generating enough streamlines to achieve robust coverage of plausible connections, which is typically on the order of millions of streamlines (Roman et al., 2019).

Finally, both deterministic and probabilistic algorithms can be improved by integrating information from a spatial extent beyond individual ODFs. In the case of probabilistic tractography, this is done by probing candidate streamline paths before committing to the next step (Aydogan & Shi, 2021; Girard et al., 2014; Tournier et al., 2010). These candidate paths can be precalculated curves or a series of steps. In deterministic tractography, one example is Tensorlines (Weinstein et al., 1999), which determines step direction by weighting the ODF information against the previous step direction, achieving momentum to propagate through voxels with less directional information.

The necessity for large numbers of streamlines lends an advantage to massively parallelized computing, and massive parallelization is straightforward: each streamline is generated independently, so the problem is embarrassingly parallel. As seen in Table 2, all recent massively parallelized local tractography implementations target the GPU. This is attributable to three reasons. (1) In most datasets, the entire white matter ODF field can be fit on a single GPU, with sufficient memory left over for tracking. (2) Each streamline can be generated independently, with each GPU thread-group (see Section 3.1) processing one streamline at a time. The size of the thread-group (32–64 threads) works well when processing ODFs, which typically have hundreds of different orientations. (3) Local tractography is best accelerated when memory is shared across cores, because each process (or, in this case, each thread-group) can refer to the same whole-brain field of ODF in memory. This contrasts with multiprocessing, where the ODFs have to be copied across all nodes.

**Table 2.** Local Tractography parallelized on GPUs and HPC systems, sorted by year, since 2015.

Authors	Year	Algorithm	Tier	Tool(s)
Hernandez-Fernandez et al.	2019	Probabilistic	1	CUDA
Shin-Ting et al.	2023	Tensorline	1	OpenGL
Kruper et al.	2025	Probabilistic	1	CUDA, OpenMP
Aeschlimann et al.	2025	Probabilistic	1	OpenCL

However, this paradigm may change in the future because sub-millimeter datasets far exceed GPU memory capacity. Such high-resolution data will require either GPU–CPU memory mapping or more advanced algorithms that distribute computation across multiple GPUs or CPUs while preserving locality in memory access.

While the first half of Hernandez-Fernandez et al. (2019) introduced GPU-accelerated ODF reconstruction, the second half introduces a CUDA-based framework for probabilistic tractography, Probtrackx. The software supports numerical integration using Euler’s method or the 2nd order Runge-Kutta method (Basser et al., 2000) to produce more stable trajectories. It also supports ROI-based propagation criteria and anatomical constraints, with the option to use meshes for surface-based seeding and termination masks. These can be used to filter streamlines during tracking, reducing the amount of resulting data needed to be transferred from the GPU. In addition to introducing these features, they performed tractography on high-resolution data with 1.35 mm isotropic voxels acquired in 270 direction/b-value combinations, generating a dense connectome from 912.82 million streamlines in an average of 2.95 hours on a single NVIDIA K80 GPU.

GPU-accelerated tractography offers significant advantages for clinical use, particularly in neurosurgical planning and intraoperative navigation. By allowing the rapid reconstruction and visualization of white-matter tracts, clinicians can assess the spatial relationships between critical fiber pathways and lesions such as tumors in real time. This capability enables interactive adjustment of regions of interest (ROIs) to refine reconstructions and immediately observe the effects—improving confidence in distinguishing displaced, infiltrated, or intact tracts. Such GPU-based interactivity reduces turnaround time between imaging and decision-making.

To demonstrate how surgeons could determine the displacement of the tract by the tumor, Wu et al. (2025; Shin-Ting et al., 2023) implemented GPU-accelerated tractography in Tensorlines with on-the-fly ROI filtering. It is based on the OpenGL pipeline. OpenGL is a cross-language, cross-platform library for rendering 2D and 3D graphics. To implement tractography via a graphical library, calculations need to be reframed into OpenGL graphical concepts. Diffusion tensors, which contain six unique values, are packed into two 3D RGB textures; seed points are treated as vertices; and streamline propagation is implemented using geometry shaders. This approach ensures portability, as OpenGL remains one of the most universally supported GPU libraries. However, because it was designed for graphics rather than general-purpose computation, implementing tractography within this framework can make the code conceptually convoluted compared to OpenCL- or CUDA-based approaches. Additionally, because the Tensorlines algorithm is deterministic, its coverage of the white matter is limited relative to probabilistic algorithms. To address this, Shin-Ting et al. used piecewise reconstruction from multiple seed ROIs. They demonstrated this technique on the fanning of the corticospinal tract, which is known to be challenging to reconstruct fully (Schilling, Blaber, et al., 2019). They reconstructed it in approximately 1 second.

Recently, Kruper, Bisson, et al. (2025) introduced a CUDA-based implementation of GPU-accelerated probabilistic tractography. This implementation integrates with the Diffusion Imaging in Python (DIPY), an open-source software library for modeling, reconstruction, and analysis of diffusion MRI data (Garyfallidis et al., 2014). DIPY provides standardized and modular implementations of common algorithms for preprocessing, denoising, diffusion modeling, fiber orientation estimation, tractography, and tractometry, and GPU-accelerated tractography can be run on any ODF supported by DIPY, such as CSD, MSMT-CSD, Q-ball, and RUMBA, among others. Written in CUDA C++ with a Python API, the software mirrors the DIPY interface and can be installed via Python’s standard package installer, pip. In terms of reproducibility, 28 major tracts recognized from the GPU-based tractography exhibit near-identical correspondence to their CPU counterparts (weighted Dice ≥ 0.94; Kruper, Bisson, et al., 2025). On average, it is able to generate over 100 million streamlines in just over an hour using the NVIDIA A40 GPU (Kruper, Bisson, et al., 2025) on data with 2 mm isotropic voxels acquired in 150 directions. It also provides the option to use multi-GPU setups for additional parallelization.

Finally, Aeschlimann et al. introduced an OpenCL-based implementation of GPU-accelerated probabilistic tractography (Aeschlimann et al., 2025). It uses PyOpenCL (see Section 3.1) and is pip-installable. Because it is based on OpenCL, it is not limited to NVIDIA GPUs. They used this implementation to generate 500 billion streamlines on data from HCP. The same group also maintains a Julia package for tractography (see Section 5.3.2 below).

### 4.3 Global and geodesic tractography

Global tractography represents a fundamentally different approach from local methods: rather than reconstructing streamlines one by one, it estimates all fibers simultaneously as part of a single optimization problem. In particle-based frameworks, short, oriented line segments called spin-glasses represent small portions of fibers within the white matter. Each spin-glass can connect to nearby segments, and their collective arrangement—evolving through probabilistic moves such as creation, deletion, and rotation—defines the global fiber configuration.

Although computationally more demanding than local methods, global tractography can be more robust to noise and local ambiguities. In one of the first public benchmarking competitions using tractography on synthetic data (Fillard et al., 2011), global tractography achieved the best scores for all metrics (Reisert et al., 2009). However, it is also much slower; run-times vary from several hours to a day (Reisert et al., 2011). Additionally, the result is sensitive to parameter choices, requiring multiple runs for adequate tuning.

Alternatively, geodesic approaches formulate tractography as a variational optimization problem, where the optimal path minimizes an integral cost function across space. The ODF field defines a spatially varying Riemannian manifold in which distances reflect local fiber orientations and densities inferred from the ODF, such that fibers correspond to geodesics, or minimal-cost trajectories, through this field. These approaches are often not as computationally demanding as fully global approaches.

Only two papers published since 2015 implemented non-local tractography on GPUs or HPC systems (see Table 3). First, Hamamci (2020), introduced a GPU-based framework for geodesic tractography formulated using cellular automata. In this approach, each voxel acts as a cell that iteratively updates its connectivity state based on its local neighborhood, propagating information through an ODF-derived cost field until convergence. This update rule is mathematically equivalent to a Bellman–Ford search for geodesics (Kauffmann & Piché, 2010), but its local nature allows full parallelization across the 3D lattice of cells. Implemented in OpenCL, the method fits cellular automata (CA) tractography on HCP subjects in an average of 0.374 seconds per subject using the NVIDIA Tesla K80 GPU.

**Table 3.** Global tractography parallelized on GPUs and HPC systems, sorted by year, since 2015.

Authors	Year	Technique	Tier	Programming model(s)
Hamamci	2020	Geodesic	1	OpenCL
Legeay	2025	Particle-based	3	Kokkos, MPI

Moving away from geodesic formulations, Legeay (2025) extended particle-based global tractography to supercomputers (Legeay, 2025). Partitions are static, with minimal communication until the final reconciliation step. The only ongoing communication involves small overlap regions at partition boundaries, which duplicate neighboring data and periodically exchange updates to maintain consistency across partitions. Parallelization is implemented using MPI for distributed execution and Kokkos for parallelization within nodes. The efficiency of this approach allowed the framework to be applied across a broad range of datasets. It successfully processed the 1,065 subjects from the Human Connectome Project in under 24 hours (using 22,784 cores and twice as many threads), reconstructed a post-mortem macaque brain at 250 *μ*m isotropic resolution, and generated a 100 *μ*m isotropic human brain reconstruction in approximately 50 hours using a large-scale supercomputing system. Taken together, this software highlights the defining strengths of supercomputers as applied to diffusion MRI. It enables the analysis of large cohorts, computationally intensive algorithms, and ultra-high-resolution data within practical time scales.

### 4.4 Streamline post-processing

Probabilistic tractography approaches, while considered highly sensitive, are also known to include many anatomically implausible connections, which can be addressed through additional post-processing steps. As seen in Table 4, there are four different post-processing approaches that have been massively parallelized. The first is tract recognition, or labeling, in which streamlines are assigned to known white matter tracts using anatomical priors. These priors typically take one of two forms: (1) ROIs, which constrain where a streamline can pass in the volume, or (2) tract atlases, where streamlines must follow trajectories similar to those in the atlas. In Yoo et al. (2015), labeling was massively parallelized using an atlas-based approach in which each streamline is represented as a piecewise linear curve parameterized by a fixed number of sample points, embedding every tract in a high-dimensional Euclidean space. Similarity between tracts was computed as the Euclidean distance between these vectors, allowing massively parallel comparison on GPU hardware. To account for inter-subject variability, the method adopts a multi-atlas framework: rather than merging all training subjects into a single atlas, a separate atlas is maintained per subject, and labeling decisions are aggregated through majority voting. Streamlines are further grouped by shape similarity, enabling simultaneous labeling of tract clusters instead of individual streamlines. This batch labeling strategy, combined with CUDA implementation, labeled approximately 1.5 million streamlines in 19 seconds using a NVIDIA GeForce GTX460 GPU.

**Table 4.** Post-processing parallelized on GPUs and HPC systems, sorted by year.

Authors	Year	Type	Tier	Programming model(s)
Yoo et al.	2015	Labeling	1	CUDA
Labra et al.	2017	Labeling	1, 2	CUDA, OpenMP
Gugnani et al.	2017	LiFE	2	MPI, OpenMP
Hernandez-Gutierrez et al.	2019	COMMIT	1	CUDA
Aggarwal & Bondhugula	2020	LiFE	1, 2	CUDA
Goicovich et al.	2021	Clustering	1	CUDA
Sreenivasan et al.	2022	LiFE	1	CUDA, HIPIFY

Similarly, Labra et al. (2017) introduced a fast atlas-based segmentation algorithm designed explicitly for massive tractography datasets. Like Yoo et al. (2015), their approach classifies streamlines by comparing them to labeled centroids in a multi-subject bundle atlas. However, it does not parameterize streamlines; instead, it uses a hierarchical “fast discard” strategy that prunes streamline–centroid comparisons using progressively simpler distance metrics. Each stage uses a subset of streamline points to eliminate implausible candidates before computing the full normalized Euclidean distance, ensuring that no valid matches are lost. They provided both a multicore version using OpenMP and a GPU version using CUDA. For the example dataset of 9 million streamlines on diffusion data with 1.7 mm isotropic resolution and 60 directions, the fastest version was the GPU version, at 5 seconds on the NVIDIA Tesla K40c.

Complementary to labeling, tractography can be filtered by evaluating it with respect to the dMRI data. Pioneering this approach, Sherbondy et al. (2009) introduced the BlueMatter algorithm and implemented it on IBM’s 2048-processor BlueGene/L supercomputer. BlueMatter evaluated a candidate set of 180 billion fascicles drawn from multiple tractography methods. The algorithm filtered streamlines to jointly minimize diffusion-prediction error and enforce white-matter volume conservation, using a distributed stochastic hill-climbing search with multiple restarts. The results exhibited all the hallmarks of successful tract filtering: accurate recovery of synthetic phantom data, broad coverage of corpus callosum pathways, and full reconstruction of Meyer’s loop within the optic radiation.

More recent algorithms for tract filtering based on match to the signal include LiFE (Linear Fascicle Evaluation) (Pestilli et al., 2014), COMMIT (Convex Optimization Modeling for Microstructure Informed Tractography) (Daducci, Dal Palù, et al., 2015), and SIFT (Spherical-deconvolution Informed Filtering of Tractograms) (Smith et al., 2013, 2015). Of these, LiFE and COMMIT have massively parallelized versions (Aggarwal & Bondhugula, 2020; Gugnani et al., 2017; Hernandez-Gutierrez et al., 2019; Sreenivasan et al., 2022). LiFE has several massively parallelized versions, in part due to the large, sparse matrix–vector multiplications that dominate its implementation. Gugnani et al. introduced a version for CPU clusters using MPI + OpenMP (Gugnani et al., 2017). Using Broadwell processors and 64 MPI processes, they fit LiFE in 3.3 minutes, though they did not provide details on the tractography algorithm used. Sreenivasan et al. introduced a GPU version using CUDA (Sreenivasan et al., 2022). It was able to fit LIFE on 1.5 million streamlines in less than 6 minutes for three different datasets, using the NVIDIA GeForce GTX 1080 Ti GPU, with similar times for the AMD Radeon RX 580 GPU. Aggarwal and Bondhugula optimized both the CPU and GPU versions (Aggarwal & Bondhugula, 2020). COMMIT has likewise been extended to take advantage of GPU acceleration. Hernandez-Gutierrez et al. (2019) fit COMMIT with the Cylinder-Zeppelin Ball multi-compartment model in 5.5 hours on 3 million streamlines using the NVIDIA Quadro P6000 GPU (Panagiotaki et al., 2012).

Finally, clustering is an unsupervised post-processing method that partitions data into groups such that streamlines within a cluster are highly similar, while those in different clusters are dissimilar. Clustered streamlines can be labeled to build new atlases or matched against existing ones. Vázquez et al. (2020) implemented a tractography clustering algorithm in both Python and C, using OpenMP to parallelize computation across threads. In this algorithm, streamlines are first broken down into a series of nodes that are clustered using K-means. Then, streamlines are grouped into preliminary fiber clusters based on their nodes, and these preliminary clusters are steadily merged into larger clusters based on a series of criteria. Goicovich et al. later extended this approach to GPUs using CUDA (Goicovich et al., 2021). They leveraged existing parallel K-means variants implemented in CUDA to test different clustering schemes and employed the CUDA Thrust library and the MapReduce paradigm to efficiently assign streamlines to preliminary clusters in parallel (Bell & Hoberock, 2012; Dean & Ghemawat, 2008; McQueen, 1967). Optimizations reduced computation time to roughly 3.5 seconds per million fibers.

### 4.5 Software availability

In Table 5, we list all software since 2015 for massively parallelized tractography based on the criteria listed in Section 2.1. This excludes software focused solely on single CPU acceleration, neural networks, or visualization. Yoo et al. (2015) and Labra et al. (2017) were excluded because we could not find the associated code online. In contrast to work published before 2015, most HPC and GPU tractography papers since have made their source code publicly accessible online. This represents a positive development for the field and a significant step toward reproducible and transparent research.

**Table 5.** Software metadata for massively parallel tractography tools.

First Author	Software Name	Available at	Published	Last Updated
Aeschlimann	tractography	https://gitlab.inria.fr/cronos/software/tractography https://github.com/cronos-inria/Tractography.jl	2025	2026
Aggarwal	Life-opt	https://github.com/karanaggarwal1994/life-opt	2020	2019
Garcıá Blas	Phardi	https://github.com/arcosuc3m/phardi	2016	2018
Goicovich	CeFFC^a^	http://www.inf.udec.cl/∼chernand/sources/CeFFC/CeFFC_sources/	2021	2021
Gugnani	MPI-LiFE	https://github.com/shashankgugnani/encode	2017	2019
Hamamci	CATractography	https://github.com/andachamamci/CATractography	2020	2022
Harms	MDT^b^	https://github.com/robbert-harms/MDT	2017	2024
Hernandez-Fernandez	cuDIMOT^c^	https://github.com/SPMIC-UoN/cudimot	2019	2025
Hernandez-Gutierrez	COMMIT^d^	https://github.com/daducci/COMMIT/pull/83	2019	2022
Xu	MATI^e^	https://github.com/jzxu0622/mati	2025	2025
Kruper	GPUStreamlines	https://github.com/dipy/GPUStreamlines	2025	2026
Legeay	ExaTract	https://framagit.org/cpoupon/gkg	2025	2025
Poirier	aodf-toolkit	https://github.com/CHrlS98/aodf-toolkit	2024	2024
Shin-Ting	VMTK-Neuro	https://www.dca.fee.unicamp.br/projects/mtk/vmtk-neuro-versions/v4.0/index.html	2023	2024
Sreenivasan	ReAl-LiFE	https://codeocean.com/capsule/7377624/tree/v1	2022	2022

a CeFFC = CUDA enhanced Fast Fiber Clustering.

b MDT = Microstructure Diffusion Toolbox.

c cuDIMOT = CUDA Diffusion Modelling Toolbox.

d COMMIT link is to CUDA port, not main branch.

e MATI = Microstructural Analysis Toolbox for Imaging.

While the tools listed in Table 5 are largely open-source, active maintenance status varies. Long-term software sustainability remains a significant challenge; the lack of dedicated funding and professional incentives for maintenance frequently results in technical debt, potentially rendering specialized high-performance tools obsolete or unusable (Connolly et al., 2023). Most tools exhibit a limited active maintenance window of 2 to 3 years following their primary publication. Two notable exceptions are the Microstructure Diffusion Toolbox (MDT), published in 2017, and cuDIMOT, published in 2019, which are both still maintained as of this writing. All software can benefit from providing containerized environments such as Docker or Singularity, as these allow reproducible deployment even years after publishing. This is particularly important for massively parallel neuroimaging pipelines because they can require specific versions of system drivers alongside their neuroimaging dependencies.

## 5 Future Work

The previous decade has achieved large speedups on existing algorithms that once formed the computational bottlenecks of tractography pipelines. This work will continue, but focus could also shift to developing new algorithms that actively leverage massive parallelization to improve accuracy rather than simply reducing runtime. As massively parallel methods mature, they create the opportunity to rethink tractography itself—integrating spatial, anatomical, and biophysical constraints that were previously computationally prohibitive. Equally important will be the commitment to standardized validation, ensuring that massively parallel methods can be rigorously compared and benchmarked.

### 5.1 Standards for massively parallel tractography

As massively parallel tractography matures, shared conventions for reporting, evaluation, and data handling would meaningfully improve rigor and comparability across studies. We discuss such standards below, concluding with a checklist for developers.

#### 5.1.1 Validation

Evaluating the performance gains of massively parallel tractography algorithms in a standardized and accessible way remains challenging. Different research groups operate under varying constraints, with access to different hardware, acquisition protocols, and scientific objectives. Consequently, reported speedups and accuracies are often difficult to compare across studies. Nonetheless, within this review, one can appreciate the scale of achievable speedups across different approaches. Moving forward, several steps could help standardize these comparisons and clarify how much of a reported gain stems from algorithmic innovation versus hardware advantage.

First, reproducible benchmarks require standardized datasets and tasks. Two datasets are already widely used in the literature: (1) The FiberCup phantom (Fillard et al., 2011) is a synthetic diffusion MRI phantom with a known ground truth of white matter fiber pathways, designed to quantitatively evaluate and compare diffusion models and tractography algorithms. Such phantoms are valuable for isolating and studying specific challenges—such as gyral bias or fiber crossings—under controlled conditions. However, subsequent studies have shown that algorithms often perform better on synthetic phantoms than on *ex vivo* or *in vivo* data, largely because phantoms simplify biological complexity (Schilling, Daducci, et al., 2019). Ongoing work aims to bridge this gap, with datasets such as 3D-VoTEM, which include both *ex vivo* brain specimens and physical phantoms, providing more realistic reference standards (Schilling, Nath, et al., 2019). (2) In contrast, the Human Connectome Project (Van Essen et al., 2012) provides a high-quality *in vivo* dataset with high angular and spatial resolution, long acquisition times, and optimized reconstruction pipelines. For algorithms targeting even higher resolutions, the submillimeter-resolution dataset from Wang et al. provides a publicly available benchmark (Wang et al., 2021). While these provide an idealized testbed for algorithmic comparisons, they may not reflect the data quality encountered in most clinical or research settings. As a result, many studies use in-house acquisitions, which vary in resolution, number of directions, and signal-to-noise ratio, hindering reproducibility and comparability.

Furthermore, there can be interactions between hardware, algorithms, and scientific interpretations. Acceleration is not a neutral process. Decisions made for computational expedience—for the massively parallelized compute systems described here but also in more standard settings—may leak into the inferences made, and this needs to be carefully evaluated and mitigated. This means that massively parallelized implementations need to be evaluated not only in terms of the speedup they provide, but also in terms of their ability to reproduce reference implementations. Thus far, authors have been successful in reproducing results, as seen in anatomical overlap scores measured using Dice-Sørensen coefficients or the Kolmogorov-Smirnov test (Kim et al., 2022; Kruper, Bisson, et al., 2025). Future studies would benefit from quantifying reproducibility using these anatomical overlap scores.

Finally, standardized validation should also extend to the outputs of tractography. Reference databases of tractography, bundles, and derived connectivity measures allow new methods to be compared against established baselines. Several such projects exist for different datasets and algorithms (Kruper et al., 2024; Li et al., 2024; Rosen & Halgren, 2021; Yeh, 2022). The most comprehensive of these is TractoInferno, a multi-site, open-access database that includes both research-grade and clinical-like acquisitions and reference tractography from four different algorithms (Poulin et al., 2022). All of these databases use local tractography; an analogous reference resource for global or geodesic tractography does not exist and is needed to validate future developments.

#### 5.1.2 File formats

The standardization of tractography file formats is an important step towards reliable, reproducible, and robust tractography (Legarreta et al., 2026). Of particular relevance to massively parallel tractography, the TRX file format was recently proposed as a standard for storing tractography data (Rheault et al., 2022) (https://tee-ar-ex.github.io). It integrates features from existing tractography formats while also introducing new capabilities. TRX supports random access with minimal memory overhead. It achieves this through a data-and-offsets design—similar to VTK9 (Schroeder et al., 1998)—in which streamline coordinates are stored as raw binary arrays, accompanied by offset tables that define streamline boundaries. This structure enables memory mapping and other low-level operations without requiring the full dataset to be loaded into RAM. These capabilities are important for handling modern tractographies that can reach hundreds of gigabytes in size and are crucial for massively parallel tractography implementations, which may otherwise become memory-bound. While broad adoption of TRX is still in its infancy, at least one implementation of massively parallelized already leveraged these features (Kruper, Bisson, et al., 2025), alongside several single-CPU implementations (Aydogan & Shi, 2021; Cieslak et al., 2021; Garyfallidis et al., 2014; Hayashi et al., 2024; Kruper, Richie-Halford, et al., 2025; Rorden, 2025; F. Zhang et al., 2020).

#### 5.1.3 Storage cost and reproducibility

Large datasets and tractographies require large amounts of disk space. Additionally, under FAIR (findable, accessible, interoperable, and reusable) principles (Wilkinson et al., 2016), intermediate outputs such as ODFs, masks, and registration results should be saved to ensure reproducibility. Collectively, these storage requirements can become substantial. When using millions of streamlines, tractography is usually the largest data derivative to store.

There are a few strategies one can employ to mitigate this cost. One is to use compression. TRX, mentioned above, includes a built-in zip compression option to reduce storage costs (Rheault et al., 2022). It also enables optional 2-byte half-floats storage, reducing total disk space to half that of more commonly used 32-bit floats. This was employed to store tractography results from HCP, with negligible effects on downstream tractometry derivatives (Kruper et al., 2024). Fiber-specific compression techniques (Presseau et al., 2015) include linearization, quantization, and an encoding step. Presseau et al. (2015) found a compression factor of over 96% for a maximum error of 0.1 mm. This compression algorithm is implemented in the Scilpy software library (Renauld et al., 2026), as well as in DIPY (Garyfallidis et al., 2014).

Since tractography can be performed more rapidly with massively parallel approaches, it may be sufficient to save the tracking parameters, randomization seeds, and software version used to generate a tractography, and not need to save the tractography results. If this approach is taken, there are a few additional considerations. Parameters should be saved in a machine-readable format and associated with a Persistent Identifier (PID). The software should be made openly available, either in a container or on a specific commit hash from a public repository. Finally, tractography derivatives should be stored and shared in place of the larger tractography.

Warrington et al. (2025) demonstrate a scalable approach to addressing these computational demands by integrating their ultra-high-field (10.5 T) diffusion MRI study with the Brainlife platform (Hayashi et al., 2024). On Brainlife, they host the raw and minimally processed data alongside containerized workflows, without storing the entire tractography. This integration automates provenance tracking and adheres to FAIR principles. They also provide their code and containers on GitHub, along with a link to the cuDIMOT GitHub page, which was used for ODF reconstruction on the GPU.

#### 5.1.4 A checklist for developers

The preceding subsections highlight a range of considerations for developing massively parallel tractography software. To consolidate these into actionable guidance, we propose the following checklist.

- To properly contextualize timing, report exact hardware specifications. This includes GPU or CPU model(s) and the number of cores or nodes used. For tier 2 and 3 compute systems, this should include details about CPU clock speed and interconnect type(s).
- Beyond hardware, additional information about the data and model is needed to contextualize timing. This should include: dataset spatial resolution, the number of diffusion volumes, the number of streamlines generated or seeds used, seeding strategy, the mean streamline length, step size, and the ODF resolution.
- At minimum, evaluate performance on at least one publicly available *in vivo* dataset. HCP is a commonly-used reference point, but its high angular and spatial resolution exceeds what is encountered in most clinical or research settings (Van Essen et al., 2012). The Healthy Brain Network (HBN) is a more representative option (Alexander et al., 2017). It is an easy-to-use reference point because it is preprocessed (Richie-Halford et al., 2022), cloud-accessible, and has a relatively lightweight access process. For submillimeter data, Wang et al. can be used (Wang et al., 2021). For additional quantitative evaluation, phantom datasets such as FiberCup can supplement *in vivo* data (Fillard et al., 2011). Smaller datasets can complement these benchmarks, but should be made publicly available to support reproducibility. See Section 5.1.1 for more discussion of available datasets.
- Provide TRX support or plan a path towards its support. For more discussion of TRX, see Section 5.1.2.
- Make results open access. If only a subset of results are able to be made public, provide a containerized script with clear instructions. More discussion on this can be found in Section 5.1.3.
- Validate reproducibility against existing methods using anatomical overlap scores such as Dice-Sørensen coefficients or the Kolmogorov-Smirnov test.

### 5.2 Design considerations

While massive parallelization offers significant theoretical speedups for tractography, practical performance can be constrained by resource allocation policies and queue dynamics. Institutional clusters can maintain separate job queues for CPU and GPU resources, each with different availability and waiting times. Transitions can introduce substantial idle time, negating any potential speed-ups.

Although these delays cannot be fully eliminated, they can be mitigated using modular pipelines. There are already multiple workflow management systems to construct and run modular pipelines. One prominent example is TractoFlow (Theaud et al., 2020), a fully automatic tractography pipeline. It integrates various neuroimaging software packages and uses the Brain Imaging Data Structure (BIDS) to ensure data interoperability and modularity (Gorgolewski et al., 2016). It is built on Nextflow, which is a pipeline creation tool that allows for parallel execution across multiple subjects and optimized resource allocation by configuring the hardware needed for each process (Di Tommaso et al., 2017). A further benefit of modularity is that it facilitates the use of pre-emptible compute, which can reduce costs. By decomposing long-running analyses into smaller, restartable modules, pipelines can leverage lower-priority queues and be more flexible to scheduling constraints than one long, monolithic process.

Additionally, maintaining memory locality and reducing memory transfer are key to preserving performance across many types of compute systems. The pattern in which data is read from and written to memory can have a dramatic impact on performance, particularly in GPU-accelerated software. In general, accessing data stored close together in memory is faster because it keeps frequently used values within fast caches and avoids repeated trips to slower global memory. On GPUs, threads within a thread-group achieve maximum efficiency when they access adjacent memory locations—known as coalesced access. Another consideration is memory transfer between the GPU and its host: these copies often traverse a relatively slow interconnect (e.g., PCIe) and can dominate runtime if not minimized or overlapped with computation.

The same principle extends to distributed systems and clusters. Here, locality refers not just to cache or device memory, but also to data placement across nodes. Computations that minimize inter-node communication and keep data close to the processors that use it—through domain decomposition, message aggregation, or asynchronous I/O—can achieve orders of magnitude better scaling. Efficient data locality is essential to ensure that computation is not bottlenecked by data transfer in multi-node systems.

Another pipeline of particular relevance is MaPPeRTrac (Cai et al., 2024). MaPPeRTrac implements a massively parallel, edge-centric pipeline that automates the transition from raw structural and diffusion MRI data to edge density images (EDI) and structural connectomes. It leverages Bedpostx (Hernández et al., 2013) and Probtrackx (Hernandez-Fernandez et al., 2019) to perform ODF reconstruction and tractography, respectively. Both of these can be run with the GPU, as discussed above. MaPPeRTrac used Parsl for parallel scripting, which allows for dynamic allocation of resources (Babuji et al., 2019). It was tested on two tier 3 and three tier 2 systems, demonstrating portability across different compute architectures.

For smaller tier 2 compute systems, Neuroimaging in Python Pipeline and Interfaces (Nipype), an open-source, community-developed software package and scriptable library that includes bindings to many major neuroimaging tools (Gorgolewski et al., 2011), can be used. Nipype offers support for HPC schedulers, but Nipype alone does not support dynamic resource allocation like Parsl or Nextflow. Thus, it could incur significant resource idling for larger workflows.

### 5.3 When to write code for the GPU

While this review has highlighted numerous software packages for GPU-accelerated tractography, a critical practical question remains: under what conditions should researchers develop new GPU-based software? Several architectural and algorithmic factors should guide this decision.

First, comprehensive tractography pipelines often incorporate a diverse array of configuration options and parameters—minor algorithmic adjustments that are typically already well-handled by existing CPU-based frameworks. Reimplementing every permutation of these options for the GPU can be an inefficient use of development resources. Instead, efforts should be concentrated on the most computationally intensive kernels within the pipeline. As previously discussed (see Section 3.1), various tools now exist to facilitate interoperability between CPU and GPU codebases, enabling a targeted approach to GPU-acceleration where only the bottlenecks are offloaded to the GPU. For Python in particular, four tools are discussed in detail below in Section 5.3.1.

Second, GPU efficiency is highly sensitive to the predictability of data access patterns. While structured memory access benefits all architectures, it is essential for achieving high throughput on GPUs through memory coalescing (see Section 3.1). ODF reconstruction generally adheres to these predictable patterns, but local tractography frequently involves more stochastic memory access as streamlines propagate through the ODF field. The performance degradation caused by such irregularity can be reduced (and even eliminated) by reducing register pressure. Lowering the register footprint per thread-group increases hardware occupancy, which allows the GPU to hide memory latency; while one thread-group waits for data to be fetched from global memory, the hardware can context-switch to another active thread-group to continue execution on the same underlying cores.

Several specific techniques can be employed to reduce register pressure in tractography algorithms. First, using single-precision floats instead of double-precision halves the register footprint. Second, leveraging the GPU’s dedicated texture memory allows interpolation on the ODFs without additional registers. Third, if available, just-in-time (JIT) compilers can be used to treat algorithm parameters as literals rather than variables. This enables the compiler to perform more aggressive optimizations and can further minimize register usage at runtime. Finally, precomputing any values that remain constant across multiple streamlines reduces thread-group register usage.

Ultimately, the choice of implementation depends on the available infrastructure. Research groups with access to high-performance GPUs can leverage these specialized algorithms for dramatic speedups, while those without such hardware can still achieve significant performance gains through optimized, multithreaded CPU implementations.

#### 5.3.1 Python and GPUs

Python has become a popular language for scientific computing (Perez et al., 2010), including in neuroimaging (Poldrack et al., 2019). Its GPU ecosystem has matured to the point where researchers can now prototype, optimize, and deploy GPU-accelerated tractography pipelines entirely in Python. To this end, we identify three Python packages particularly well-suited for neuroimaging.

The first, CuPy (Nishino & Loomis, 2017), provides a nearly drop-in GPU-accelerated replacement for NumPy and SciPy methods, making it one of the easiest entry points to GPU computing in Python. It integrates smoothly with other packages linking Python to the GPU. This may be useful for researchers who wish to quickly port a NumPy-based codebase to the GPU.

The second, CUDA Python, is maintained by NVIDIA and provides native support for compiling and executing CUDA C kernels directly from Python. It can be installed with pip and supports JIT compilation. Taken together, this facilitates direct hardware optimization via CUDA C, while retaining the portability and accessibility of Python.

Finally, Numba (Lam et al., 2015) provides a higher-level route, compiling selected Python functions to GPU kernels. While it allows code to remain largely NumPy-compatible, its syntax often converges toward CUDA C, as it ultimately targets the same CUDA hardware.

#### 5.3.2 Julia

While Python remains the dominant interface for deep learning, the Julia programming language has emerged as an alternative (Bezanson et al., 2012). It features native capabilities for compiling to GPUs, integration with the Message Passing Interface (MPI), and multi-threading. These architectural features theoretically allow a single codebase to execute across Tier 0, 1, and 2 compute systems, which reduces the effort associated with maintaining disparate codebases for different hardware targets. Similar to Python, Julia prioritizes a high-level, human-readable syntax designed for researcher accessibility. However, Python currently benefits from a larger user base, which typically correlates with a broader ecosystem of third-party packages, more active maintenance, and more comprehensive documentation.

### 5.4 Modeling complex spatial relationships

Several algorithms utilize voxel-wise spatial context to better inform tractography. While methods such as PTT, a-ODF, geodesic tractography, and iFOD2 (Tournier et al., 2019) operate within local neighborhoods, global tractography instead optimizes pathways by considering brain-wide spatial relationships.

#### 5.4.1 Neighborhood approaches

Increased computational power opens opportunities to model spatial relationships across neighboring voxels. This can be seen in the existing software to massively parallelize a-ODFs, as discussed above in Section 4.1. Another way to integrate information across voxels is within local tractography propagation. Parallel transport tractography (PTT) is a popular tractography algorithm that can generate geometrically smooth curves using parallel transport frames (Aydogan & Shi, 2021). In a recent phantom-data tractography challenge, the top three methods—ranked by how well their connectivity matched the ground truth—all used PTT (Girard et al., 2023). PTT uses a cylindrical probe to determine its next step during local tractography. A potential improvement would be to sample from a suite of more complicated probes in order to better match the underlying fiber bundle geometry. However, this is computationally more expensive, and thus could benefit from massive parallelization.

#### 5.4.2 Global approaches

The brain-wide approach of global tractography improves robustness to noise and local perturbations (Mangin et al., 2013). Yet, it remains less common than local methods due to its high computational cost relative to its perceived marginal benefits. Although runtimes can be reduced to several hours via regularization and boundary conditions (Reisert et al., 2011), these constraints may diminish the inherent advantages of the global approach. Recent work by Legeay (2025) utilized a tier 3 compute system to scale global methods to large datasets like the HCP; however, translating and demonstrating these improvements on lower-tier compute systems remains necessary for broader adoption.

Even if computational barriers are removed, the choice between global and local probabilistic tractography involves a fundamental sensitivity-specificity trade-off. Streamlines can be conceptualized as true positives (TP), false positives (FP), true negatives (TN), or false negatives (FN), depending on their correspondence to underlying groups of axons. While phantom data allows determination of which category a streamline belongs to, real-world validation relies on imperfect references such as anatomical landmarks, consistency with the dMRI data, or high-resolution multimodal comparisons. Generally, probabilistic tractography prioritizes sensitivity, reducing FNs at the expense of increased FPs. In contrast, global tractography produces fewer FPs to better balance sensitivity and specificity (Girard et al., 2020). The optimal choice of algorithm depends on the scientific question. Strict post-processing filters may tolerate the high FP rates of probabilistic tractography, whereas the direct use of a tractography to calculate connectivity metrics may benefit from a more balanced approach like global tracking.

### 5.5 Ensemble algorithms

In local tractography, there are many algorithms, and each requires the user to set several parameters. Choosing a single algorithm with specific parameters poses two challenges. First, different algorithms and parameter values produce different results. Second, the optimal choice of algorithm and parameter value may differ between white matter regions or tracts, subjects, and acquisition parameters. Over the years, a few approaches have been proposed to alleviate these issues.

One proposal to tackle this problem was ensemble tractography (Takemura et al., 2016), which proposed to systematically combine streamlines from an ensemble of algorithms (in the original implementation, both deterministic and probabilistic) with systematically varying parameters (originally, curvature and stopping criterion). Then, this large tractography was whittled down using a post-processing algorithm. LiFE was used in the original implementation, but this can also be done with other algorithms, such as COMMIT or SIFT. Using this approach, Takemura et al. showed that the benefits of different local tractography approaches could be reaped to best explain the dMRI signal. More recently, this approach was extended using streamline-specific parameters (SSP) (Vink et al., 2024). Here, each streamline is generated by sampling from a distribution of parameters and algorithms, with no two streamlines having the same parameters, resulting in a denser sampling of the parameter space.

Massively parallel tractography opens the opportunity to change our approach to local tractography. Currently, streamline selection follows a two-step approach—generation followed by filtering. However, future work could explore performing a one-step approach in which streamlines are drawn dynamically from a changing distribution. This would enable the systematic reduction of known tractography biases. Tract generation could adjust to satisfy constraints not only set by existing filtering algorithms, but also additional constraints of interest to the researchers, such as full gyral coverage, reconstruction of known white matter tracts, representation of fanning regions, or tracking through high-curvature pathways such as Meyer’s Loop. The process would terminate only once all constraints were sufficiently satisfied. Streamline filtering could occur immediately after individual streamline generation (within the node or GPU), minimizing costly memory transfers. This strategy could also be integrated into interactive pipelines, in which streamlines are generated preferentially using parameter settings that previously produced streamlines satisfying the specified constraints. Taken together, such approaches would reframe tractography as a constrained and adaptive process rather than a static generate-then-filter one.

However, increasing the parameter space introduces potential risks. Streamline generation should remain data-driven; excessive parameter flexibility alongside overly stringent criteria may cause streamlines to overfit to priors rather than the underlying diffusion signal. Selection criteria should therefore either be grounded in the diffusion data—as seen in frameworks like COMMIT, LiFE, or SIFT (Daducci, Dal Palù, et al., 2015; Pestilli et al., 2014; Smith et al., 2015)—or supported by clearly defined and well-justified anatomical priors. In the case of these filtering algorithms, a demonstration of inference stability and a formal convergence criterion would also be necessary to ensure robust results.

## 6 Conclusions

High-performance computing and graphics processing units have transformed tractography from a notoriously time-consuming process into a scalable, interactive, and experimentally flexible endeavor. Across every stage—ODF reconstruction, tract generation, global optimization, and filtering—massive parallelization has enabled both dramatic speedups and entirely new classes of algorithms, from real-time clinical visualization to exa-scale global reconstructions. New tools are also making the development of massively parallel code increasingly accessible. The speed and convenience of these advances should now be leveraged to produce tractography that is not only faster, but also more accurate.

## Ethics

Ethical approval was not required for this review as it is based solely on previously published studies.

## Data Availability

This review did not generate new data or code. All software packages discussed are publicly available at the repositories listed in Table 5.
